# Bowel Transit Time May Be Accelerated in Colonic Diverticulosis Independent of Mucosal Serotonin Signaling

**DOI:** 10.3390/jcm14248626

**Published:** 2025-12-05

**Authors:** Piotr Nehring, Miłosz Jastrzębski, Ilona Joniec-Maciejak, Adriana Wawer, Adam Przybyłkowski

**Affiliations:** 1 Department of Gastroenterology and Internal Medicine, Medical University of Warsaw, Banacha 1a, 02-097 Warsaw, Poland; piotr.nehring@wum.edu.pl (P.N.);; 2 Department of Experimental and Clinical Pharmacology, Centre for Preclinical Research and Technology, Medical University of Warsaw, ul. Żwirki i Wigury 61, 02-091 Warsaw, Poland

**Keywords:** biogenic amines, colonic diverticulosis, serotonin, time, transit

## Abstract

**Background/Objectives**: Colonic diverticulosis is a common condition in the elderly population and may affect bowel habits and reduce quality of life. Intestinal peristalsis is regulated by bioamines, which can influence bowel transit time. **Methods**: This prospective, comparative study included 23 patients. All participants were examined with colonoscopy with colonic mucosal biopsy and a bowel transit time test using SITZMARKS^®^ markers. The following bioamines were assessed in the colonic mucosa: 3-methoxy-4-hydroxyphenylglycol (MHPG), norepinephrine (NA), dopamine (DA), homovanillic acid (HVA), 5-hydroxytryptamine (5-HT, serotonin), and 5-hydroxyindoleacetic acid (5-HIAA). **Results**: Among study participants, 14 had colonic diverticulosis and 9 were controls. There were no differences in age, sex, body mass, or weight between the groups. All patients with diverticulosis had left-sided diverticula and a DICA score of 1. None of the patients met the diagnostic criteria outlined in the ROME IV classification of functional gastrointestinal disorders. After 48 h, patients with diverticulosis tended to retain less SITZMARKS^®^ markers (mean of 1.14 vs. 6.78, *p* < 0.027), compared to the control group. Fewer patients with diverticulosis tended to have SITZMARKS^®^ markers visible in the X-ray image at 48 h (2 out of 14 versus 4 out of 9 patients, *p* < 0.262), compared to the control group. There were no differences in colonic mucosal concentrations of bioamines (5-HT, 5-HIAA, MHPG, NA, DA, HVA) between cases and controls. **Conclusions**: Bowel transit time in patients with colonic diverticulosis may be accelerated compared with controls, and this appears unrelated to bioamine metabolism.

## 1. Introduction

Digestive diseases are common worldwide, affecting roughly 15–20% of the global population. Colonic diverticulosis refers to the formation of pouch-like protrusions that emerge where the colonic wall is structurally vulnerable. These outpouchings involve herniation of the inner mucosal layers through weakened regions of the muscularis. The likelihood of developing diverticulosis rises substantially over the lifespan. Although relatively uncommon in younger adults, it becomes widespread in older populations in Western countries, up to 70% or more after 80 years. By contrast, its occurrence is considerably lower in many Asian and African regions, where demographic, dietary, and environmental factors differ [1]. While diverticulosis is most commonly observed in the sigmoid colon, geographic and ethnic variations exist, with a lower prevalence in Asian populations where diverticula are predominantly right-sided [2]. The pathophysiology involves structural weakness of the colonic wall, increased intraluminal pressure, altered motility, and environmental factors such as low-fiber diets, obesity, and sedentary lifestyle; genetic and microbiota changes also contribute [3,4]. Management of asymptomatic diverticulosis focuses on dietary fiber and lifestyle modification, while complicated cases may require antibiotics, bowel rest, and, in severe or recurrent episodes, surgical intervention.

Colonic motility plays a crucial role in the pathogenesis of diverticulosis, and the bowel transit time (BTT) serves as a key functional marker. Delayed BTT has been reported in patients with diverticulosis, particularly in those with left-sided diverticula [5]. Slower transit times may increase intraluminal pressure and contribute to diverticula formation by enhancing strain on the colonic wall [6]. Serotonin plays a central regulatory role in gastrointestinal motor activity and sensory signaling. Most of the body’s serotonin is produced locally in the gut by enterochromaffin cells, which release it in response to mechanical and chemical cues to coordinate reflex pathways that control motility [7,8]. In a motility study by Sugihara et al., the intraluminal pressure in patients with diverticula of the right colon and controls was compared [9]. In that study, patients with diverticula presented a significantly higher intestinal motility index both at rest and after administration of neostigmine. That may indicate that increased intraluminal pressure and abnormal motility may play an important role in the pathogenesis of colonic diverticula [9].

This study aimed to assess BTT in patients with diverticulosis compared to controls and to correlate these findings with bioamine concentrations.

The rationale for conducting this study was the lack of research on the impact of diverticula on intestinal motility in colonic diverticulosis. To date, no study has evaluated this using the applied methodology.

## 2. Materials and Methods

### 2.1. Patients

This preliminary prospective study included patients who had colonoscopy at the Department of Gastroenterology and Internal Medicine, Medical University of Warsaw, Poland. All participants were inpatients and endoscopy was performed in one Endoscopy Unit. All participants completed the ROME IV questionnaire to screen for functional gastrointestinal disorders prior to the procedure. Colonic mucosal biopsies were obtained from both the right and left colon.

Participants were divided into two groups: those with left-sided colonic diverticulosis confirmed by colonoscopy, and controls (qualified after colonoscopy, detailed history and ROME IC questionnaire). The exclusion criteria for both groups included a history of intestinal resection, gastrointestinal bleeding, cancer, radiotherapy, diverticulitis, inflammatory bowel disease, irritable bowel syndrome, and the use of SSRIs, SNRIs, or pizotifen.

All participants provided informed consent, and the study was approved by the Medical University of Warsaw Ethics Committee.

### 2.2. Colonoscopy

Colonoscopy was performed under intravenous sedation using an Evis Exera III CF-HQ190L videocolonoscope (Olympus, Tokyo, Japan). Mucosal biopsies from the colon segment affected by the diverticula were obtained using biopsy forceps (Olympus, Tokyo, Japan). Colon mucosa biopsies were promptly transferred to microtubes and preserved under deep-freeze conditions to maintain biochemical integrity. The storage time of tissue samples did not exceed six months to analysis. Patients with diverticulosis were assessed using the Diverticular Inflammation and Complication Assessment (DICA) endoscopic scale [10].

### 2.3. High-Performance Liquid Chromatography

Intestinal mucosal samples were weighed, homogenized in 1000 μL of 0.05 mM ascorbic acid and 0.1 N perchloric acid using an ultrasonic disrupter, and then centrifuged at 13,000× *g* for 15 min at 4 °C. The supernatant was filtered through (0.2 μm) and analyzed via HPLC-ED with a Knauer Mini-Star pump (LCCO Biotech OÜ, Tallinn, Estonia), LaChrom autosampler, and electrochemical detector (DataApex Ltd., Dobřichovice, Czech Republic). Chromatograms were recorded using the Clarity software version 10.1.1 (DataApex Ltd., Dobřichovice, Czech Republic), with quantification based on Sigma-Aldrich standards (Sigma-Aldrich., Saint Louis, MO, USA). The following bioamines were analyzed: 3-methoxy-4-hydroxyphenylglycol (MHPG), norepinephrine (NA), dopamine (DA), homovanilic acid (HVA), 5-hydroxytryptamine (5-HT, serotonin), and 5-hydroxyindoleacetic acid (5-HIAA). All HPLC analyses were performed with a negative control.

### 2.4. Bowel Transit Time Test

A BTT test was performed two weeks after the colonoscopy. To evaluate the total gastrointestinal transit time, patients were asked to take a SITZMARKS^®^ capsule containing 24 small radiopaque markers (Konsyl Pharmaceuticals Inc., Easton, MD, USA). After a few pilot transit time tests, we observed a faster transit of the markers throughout the colon than in the protocol provided by the producer. Abdominal radiography was performed 48 h and (if needed) 96 h after capsule admission. BTT were evaluated by counting the markers that remained in the colon on the day the X-ray was performed.

### 2.5. Statistical Analysis

All quantitative variables were evaluated for normality using Kolmogorov–Smirnov and Lilliefors tests. All quantitative variables showed deviation from normal distribution and were assessed using the Mann–Whitney U test. All analyses for multiple comparisons are presented with corrections for multiple tests. Missing data were handled using pairwise deletions. Statistical significance was set at *p* < 0.05. All analyses were performed using the Statistica software version 13.3 (StatSoft, Inc., Tulsa, OK, USA).

## 3. Results

### 3.1. General Characteristics

The study included 23 patients, 14 with colonic diverticulosis, and 9 controls. There were no differences in age, sex, body mass, or weight between the groups. All patients with diverticulosis had left-sided diverticula and had a DICA score of 1. None of the patients included in the study fulfilled the diagnostic criteria outlined in the ROME IV classification of functional gastrointestinal disorders. The characteristics and comparisons of the study participants are shown in Table 1.

### 3.2. Bowel Transit Time

After 48 h, patients with diverticulosis tended to retained fewer SITZMARKS^®^ markers (mean of 1.14 vs. 6.78, *p* < 0.027), compared to the control group. Fewer patients with diverticulosis tended to have SITZMARKS^®^ markers visible in the X-ray image at 48 h (2 out of 14 versus 4 out of 9 patients, *p* < 0.262), compared to the control group.

### 3.3. Colonic Mucosa Bioamines

There were no differences in colonic mucosa concentrations of bioamines (5-HT, 5-HIAA, MHPG, NA, DA, HVA) between cases and controls (Supplementary Table S1).

## 4. Discussion

The presented findings are of a preliminary nature, and the results indicate a tendency toward altered bowel transit time in patients with diverticulosis.

The findings of this study highlight several notable differences compared to previous research on patients with colonic diverticulosis and its impact on BTT and mucosal biogenic amines. The significant alteration in BTT was expressed as a reduction in visible SITZMARKS^®^ markers in patients with diverticulosis after 48 h compared to the control group, contrary to earlier findings [11]. Our findings are in line with a recent study by Peery et al., which showed that patients with colonic diverticula are more likely to have more stools per day than controls, and this association was stronger in individuals with >10 diverticula [12]. However, Peery et al. studied data on bowel movements based only on a patient questionnaire.

In contrary to the aforementioned results there were suggestions on delayed BTT in patients with colon diverticula [5]. However, there are no direct studies comparing BTT between patients with diverticulosis and control subjects. The common, unjustified belief that patients with diverticulosis have prolonged BTT stems from an extrapolation of the association between constipation—a postulated risk factor—and the development of diverticula.

Although accelerated colonic transit in diverticulosis may appear counterintuitive, several physiological adaptations could account for it. Enhanced segmental contractility, subtle motility dysregulation, or structural remodeling of the colon may promote more rapid propulsion. These processes likely interact, but their relative contribution remains uncertain. Additionally, colonic shortening observed in advanced disease reduces the distance for transit, inherently speeding passage. These factors likely act in combination, underscoring the need for further studies integrating motility assessment and anatomical evaluation.

One of the probable explanations of increased BTT in patients with diverticulosis in the presented study may result from altered serotonergic pathways in the affected colonic segment [13]. However, in this study, contrary to authors previous research, there were no differences in bioamines concentration between cases and control, which may result from the relatively small sample size and low power of the estimations [13]. Serotonin metabolism is a promising therapeutic target in functional bowel disorders, with drugs such as 5-HT_4_ receptor agonists (e.g., prucalopride) enhancing gastrointestinal motility, and 5-HT_3_ receptor antagonists (e.g., alosetron) reducing visceral hypersensitivity and diarrhea. However, similar to other studies, we showed no differences in serotonin concentration between patients with diverticulosis and the control group [14]. Gershon et al. noted that altered serotonin signaling can lead to significant motility issues in patients with various gastrointestinal disorders. Further studies have highlighted the potential therapeutic implications of targeting serotonin receptors in managing functional gastrointestinal disorders [15,16,17].

Variation in bowel motility in patients with colonic diverticulosis may also result from changes in microbiota [18,19,20,21]. Recent assessments of the gut microbiome in individuals with diverticulosis show inconsistent findings [18]. Some investigations report subtle shifts in the abundance of specific bacterial taxa, whereas overall community diversity often appears unchanged [19]. Other studies, including those relying on mucosal sampling, suggest only minimal microbial differences between individuals with and without diverticula [20,21]. Collectively, these results indicate that, if microbiome alterations do occur, they may be modest or secondary to other disease processes. It was shown, that patients with diverticulitis may exhibit greater fecal microbiota diversity compared to control subjects from a heterogeneous population, with the phylum *Proteobacteria* emerging as the principal taxonomic group distinguishing the two cohorts [22]. It is still unclear whether changes in gut microbiota are primary or secondary to diverticulitis; moreover, bacteria’s role in diverticulosis formation seems to be complex.

Whether the observed changes in BTT among patients with diverticulosis represent a primary pathophysiological mechanism or a secondary effect of diverticular formation remains to be determined. Regardless of the plausible mechanism of altered BTT, the conflicting reports on BTT length in diverticulosis indicate a need for a personalized approach to diagnosis and treatment in patients with symptomatic colon diverticula. Evaluation of colon transit time seems essential for appropriate individualized therapeutic strategy selection. While high-fiber diets have traditionally been recommended to improve bowel transit and alleviate constipation, the relation between fiber intake and diverticulosis is more complex [23]. Some evidence suggests that fiber does not necessarily reduce the risk of diverticulosis or improve stool consistency in patients with this condition, complicating its role in transit time management. A comprehensive systematic review highlights the lack of high-quality evidence supporting its effectiveness, particularly in preventing recurrent diverticulitis [24]. Current dietary recommendations are largely based on inconsistent and low-level evidence, underscoring the need for well-designed clinical trials to clarify the role of high-fiber diets in the management of diverticular disease. A personalized approach to dietary and pharmacological management may be key to successful prevention of diverticulitis, as uniform recommendations often fail to account for individual variability in risk factors, disease presentation, and treatment response [25,26,27,28,29].

Nutritional guidance for patients with diverticular disease remains uncertain because available studies offer mixed or low-quality evidence. While increased fiber intake has traditionally been encouraged, research consistently shows that its benefits are not uniform across patients. The presented results support reconsidering restrictive dietary guidelines for diverticular disease. More individualized dietary strategies may therefore be more appropriate than generalized restrictions [30].

This is the first, however preliminary, study assessing directly bowel transit in patients with colonic diverticulosis. This is the first attempt to correlate bowel transit with serotonin metabolism.

As only mucosal bioamine concentrations were analyzed, these results may not fully represent activity within deeper layers such as the enteric nervous system or muscularis, which are key regulators of motility. The study is limited by its small cohort size, single-center design, and lack of longitudinal follow-up.

In summary, the presented study confirms some previous findings regarding altered BTT in patients with colonic diverticulosis. This could pave the way for future research focusing on alterations in diverticular disease.

## Figures and Tables

**Table 1:** Characteristics of the studied groups and comparisons.

	Diverticulosis N = 14	Controls N = 9	*p* Value
Basic Characteristics			
Age, y, mean ± SD	60.5 ± 12.9	55.3 ± 19.6	<0.434
Sex, male/female (%/%)	9/5 (64/36)	2/7 (22/78)	<0.123
Height, cm, mean ± SD	171.5 ± 10.7	169 ± 4.7	<0.520
Weight, kg, mean ± SD	79.9 ± 20.7	73.7 ± 12.4	<0.430
BMI, kg/m^2^, mean ± SD	26.9 ± 5.1	25.8 ± 4.4	<0.534
Bowel Transit Time			
Number of SITZMARKS^®^ markers after 48 h, mean ± SD	1.14 ± 2.71	6.78 ± 8.32	<0.027
Presence of SITZMARKS^®^ markers after 48 h, n (%/%)	2/12 (14/86)	4/5 (44/56)	<0.262

SD—standard deviation.

## Data Availability

The dataset is available on reasonable demand from the corresponding author.

## Supplementary Materials

The following supporting information can be downloaded at: https://www.mdpi.com/article/10.3390/jcm14248626/s1. Supplementary Table S1: Bioamine concentrations in the studied groups and comparisons.

## References

[1] Peery A.F., Crockett S.D., Barritt A.S., Dellon E.S., Eluri S., Gangarosa L.M., Jensen E.T., Lund J.L., Pasricha S., Runge T. (2015). Burden of Gastrointestinal, Liver, and Pancreatic Diseases in the United States. Gastroenterology.

[2] Nagata N., Sakamoto K., Arai T., Niikura R., Shimbo T., Shinozaki M., Aoki T., Sekine K., Okubo H., Watanabe K. (2015). Visceral fat accumulation affects risk of colonic diverticular hemorrhage. Int. J. Color. Dis..

[3] Strate L.L., Morris A.M. (2019). Epidemiology, Pathophysiology, and Treatment of Diverticulitis. Gastroenterology.

[4] Tursi A., Scarpignato C., Strate L.L., Lanas A., Kruis W., Lahat A., Danese S. (2020). Colonic diverticular disease. Nat. Rev. Dis. Primers.

[5] Andeweg C.S., Mulder I.M., Felt-Bersma R.J., Verbon A., van der Wilt G.J., van Goor H., Lange J.F., Stoker J., Boermeester M.A., Bleichrodt R.P. (2013). Guidelines of diagnostics and treatment of acute left-sided colonic diverticulitis. Dig. Surg..

[6] Piscopo N., Ellul P. (2020). Diverticular Disease: A Review on Pathophysiology and Recent Evidence. Ulster Med. J..

[7] Najjar S.A., Hung L.Y., Margolis K.G. (2023). Serotonergic Control of Gastrointestinal Development, Motility, and Inflammation. Compr. Physiol..

[8] Sikander A., Rana S.V., Prasad K.K. (2009). Role of serotonin in gastrointestinal motility and irritable bowel syndrome. Clin. Chim. Acta.

[9] Sharara A.I. (2018). Diverticular Disease of the Colon. Inflamm. Intest. Dis..

[10] Cambie G., Violi A., Miraglia C., Barchi A., Nouvenne A., Capasso M., Leandro G., Meschi T., De’ Angelis G.L., Di Mario F. (2018). Development and usefulness of the new endoscopic classification: DICA. Acta Biomed..

[11] Bassotti G., Battaglia E., De Roberto G., Morelli A., Tonini M., Villanacci V. (2005). Alterations in colonic motility and relationship to pain in colonic diverticulosis. Clin. Gastroenterol. Hepatol..

[12] Peery A.F., Keku T.O., Galanko J.A., Sandler R.S. (2022). Colonic Diverticulosis Is Not Associated With Painful Abdominal Symptoms in a US Population. Gastro Hep Adv..

[13] Jastrzebski M., Nehring P., Joniec-Maciejak I., Wawer A., Przybylkowski A. (2023). Serotonin Metabolism and Serotonin Receptors Expression Are Altered in Colon Diverticulosis. Medicina.

[14] Costedio M.M., Coates M.D., Danielson A.B., Buttolph T.R., Blaszyk H.J., Mawe G.M., Hyman N.H. (2008). Serotonin signaling in diverticular disease. J. Gastrointest. Surg..

[15] Gershon M.D., Tack J. (2007). The serotonin signaling system: From basic understanding to drug development for functional GI disorders. Gastroenterology.

[16] Neal K.B., Bornstein J.C. (2006). Serotonergic receptors in therapeutic approaches to gastrointestinal disorders. Curr. Opin. Pharmacol..

[17] Kotsiliti E. (2022). Enteric neurons and gut homeostasis. Nat. Rev. Gastroenterol. Hepatol..

[18] Hua X., McGoldrick J., Nakrour N., Staller K., Chung D.C., Xavier R.J., Khalili H. (2024). Gut microbiome structure and function in asymptomatic diverticulosis. Genome Med..

[19] Jones R.B., Fodor A.A., Peery A.F., Tsilimigras M.C.B., Winglee K., McCoy A., Sioda M., Sandler R.S., Keku T.O. (2018). An Aberrant Microbiota is not Strongly Associated with Incidental Colonic Diverticulosis. Sci. Rep..

[20] van Rossen T.M., Ooijevaar R.E., Kuyvenhoven J.P., Eck A., Bril H., Buijsman R., Boermeester M.A., Stockmann H., de Korte N., Budding A.E. (2021). Microbiota composition and mucosal immunity in patients with asymptomatic diverticulosis and controls. PLoS ONE.

[21] Alexandersson B.T., Hugerth L.W., Hedin C., Forsberg A., Talley N.J., Agreus L., Jarbrink-Sehgal E., Engstrand L., Andreasson A., Schmidt P.T. (2023). Diverticulosis is not associated with altered gut microbiota nor is it predictive of future diverticulitis: A population-based colonoscopy study. Scand. J. Gastroenterol..

[22] Daniels L., Budding A.E., de Korte N., Eck A., Bogaards J.A., Stockmann H.B., Consten E.C., Savelkoul P.H., Boermeester M.A. (2014). Fecal microbiome analysis as a diagnostic test for diverticulitis. Eur. J. Clin. Microbiol. Infect. Dis..

[23] Peery A.F., Barrett P.R., Park D., Rogers A.J., Galanko J.A., Martin C.F., Sandler R.S. (2012). A high-fiber diet does not protect against asymptomatic diverticulosis. Gastroenterology.

[24] Unlu C., Daniels L., Vrouenraets B.C., Boermeester M.A. (2012). A systematic review of high-fibre dietary therapy in diverticular disease. Int. J. Color. Dis..

[25] Rezapour M., Ali S., Stollman N. (2018). Diverticular Disease: An Update on Pathogenesis and Management. Gut Liver.

[26] Hall J.K., Supiano M.A., Cohan J.N. (2025). Diverticulitis in Older Adults: A Review of Etiology, Diagnosis, and Management. J. Am. Geriatr. Soc..

[27] Stewart D.B. (2024). Individualized care or ad hoc care: Is evidence based, personalized care for diverticulitis currently possible?. Surg Open Sci..

[28] Melio A.A., Johnson M., Kaplan J.A., Moonka R., Simianu V.V. (2024). Treatment preferences in diverticulitis are common and rarely change after a clinic visit. Surg. Open Sci..

[29] Ali Ismail A.M., Saad A.E., Fouad Abd-Elrahman N.A., Abdelhalim Elfahl A.M. (2022). Effect of Benson’s relaxation therapy alone or combined with aerobic exercise on cortisol, sleeping quality, estrogen, and severity of dyspeptic symptoms in perimenopausal women with functional dyspepsia. Eur. Rev. Med Pharmacol. Sci..

[30] Strate L.L., Liu Y.L., Syngal S., Aldoori W.H., Giovannucci E.L. (2008). Nut, corn, and popcorn consumption and the incidence of diverticular disease. JAMA.

