# Supplementary material for: Bowel Transit Time May Be Accelerated in Colonic Diverticulosis Independent of Mucosal Serotonin Signaling
Source: J Clin Med. 2025 Dec 5;14(24):8626. doi: 10.3390/jcm14248626 (PMC12733458; doi:10.3390/jcm14248626)
Supplement: Supplementary file 1 [file jcm-14-08626-s001.zip › jcm-3985289-supplementary.pdf]

Supplementary Table S1. Bioamines concentration the studied groups and comparisons.

	**Cases**<br>**N = 14**	**Controls**<br>**N = 9**	***P* value**	**Power**
Right colon				
NA, median pg/mg (Q1-Q4)	168.8 (103.1 – 223.7)	141.4 (100.1 – 188.4)	<0.469	1.00
MHPG, median pg/mg (Q1-Q4)	2.4 (0.7 – 8.7)	1.7 (0.9 – 4.5)	<0.875	0.05
DA, median pg/mg (Q1-Q4)	4.9 (1.4 – 13.1)	4.2 (0.8 – 5.6)	<0.395	0.05
HVA, median pg/mg (Q1-Q4)	2.2 (0.9 – 4.5)	7.5 (0.9 – 16.1)	<0.156	0.22
5-HT, median pg/mg (Q1-Q4)	5126 (2624 – 6947)	8185 (5523 – 9338)	<0.156	1.00
5-HIAA, median pg/mg (Q1-Q4)	2682 (1273 –3711)	2927 (1958 – 5576)	<0.508	1.00
Left colon				
NA, median pg/mg (Q1-Q4)	115.0 (81.2 – 154.9)	111.4 (96.1 – 141.7)	<0.095	0.13
MHPG, median pg/mg (Q1-Q4)	1.1 (0.7 – 6.9)	1.1 (0.6 – 2.1)	<0.682	0.05
DA, median pg/mg (Q1-Q4)	7.5 (3.8 – 10.4)	11.2 (3.0 – 17.6)	<0.271	0.13
HVA, median pg/mg (Q1-Q4)	0.9 (0.3 – 3.1)	4.3 (1.5 – 10.2)	<0.108	0.12
5-HT, median pg/mg (Q1-Q4)	7350 (4461 – 15898)	6634 (4687 – 8207)	<0.682	1.00
5-HIAA, median pg/mg (Q1-Q4)	2601 (1043 – 4095)	2200 (1544 – 4933)	<0.431	1.00

Q1-Q4 – interquartile range;
